# Sensitive electrochemical gold nanoparticle-based immunosensor for norovirus detection in food samples

**DOI:** 10.1039/d3ra08586d

**Published:** 2024-02-16

**Authors:** Paulina Janicka, Sylwia Baluta, Juliusz Winiarski, Kinga Halicka-Stępień, Aleksandra Pogorzelska, Joanna Cabaj, Katarzyna Pala, Barbara Bażanów

**Affiliations:** a Department of Pathology, University of Environmental and Life Sciences in Wrocław Norwida 31 50-375 Wrocław Poland; b Food4Future Technologies Sp. z o. o. ul. Tarasa Szewczenki 24 51-351 Wrocław Poland; c Institute of Advanced Materials, Wrocław University of Science and Technology Wybrzeże Wyspiańskiego 27 50-370 Wrocław Poland sylwia.baluta@pwr.edu.pl; d Group of Surface Technology, Department of Advanced Material Technologies, Wroclaw University of Science and Technology Wybrzeże Wyspiańskiego 27 Wroclaw 50-370 Poland; e Water Science and Technology Institute- H_2_O SciTech ul. Tarasa Szewczenki 24 51-351 Wrocław Poland

## Abstract

Norovirus (NoV) infection is one of the most common non-bacterial causes of gastroenteritis among the population worldwide. From the point of view of medical diagnostics, it is important to develop a system that would sensitively and selectively detect norovirus from a patient's sample in order to control and limit its spread. In this paper, we present a stable and sensitive NoV (mouse model) detection matrix in infected food samples. The bio-platform was made of a modified gold electrode with a self-assembled l-cysteine monolayer, covered with gold nanoparticles, a linker and an antibody specific to the VP1 surface protein of the virus. Binding of the VP1 protein to the antibody caused a decrease in the current strength confirmed by electrochemical techniques – cyclic voltammetry (CV) and differential pulse voltammetry. The reduction of the current was proportional to the concentration of NoV sample. The biosensors showed high sensitivity and linearity in a range from 1 × 10^−9^ to 1 × 10^−18^ TCID_50,_ with the detection limit of 1 × 10^−18^ TCID_50._ CV showed a diffusion-controlled process. In addition, each modification step was confirmed by scanning electron microscopy, electrochemical impedance spectroscopy, and CV. The described immunosensor showed excellent recovery values, good linearity and long-term stability, crucial parameters for biosensor construction.

## Abstract 2

Detection of Norovirus (NoV), a prevalent non-bacterial gastroenteritis agent globally, is crucial. Developing a biosensor for NoV detection is of utmost importance.

## Graphical abstract

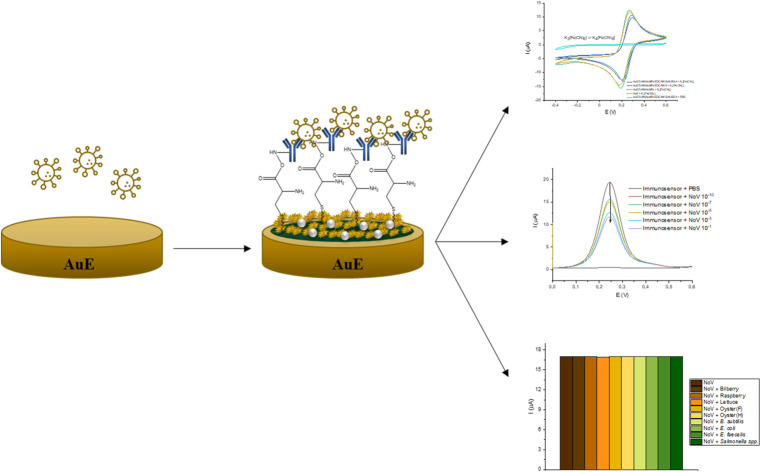

## 1. Introduction

The global pandemic caused by the SARS-CoV-2 virus has demonstrated an urgent need for non-invasive, fast, and cheap diagnostic methods for emerging pathogens.^1^ Norovirus (NoV) is one of the main aetiological agents of viral gastroenteritis, causing 685 million cases and 210 000 deaths each year.^2,3^ Norovirus infection is caused by a low amount of the virus (*c.a.* 100 copies per mL), and currently, there is no vaccine nor anti-noroviral agents, while the most common source of infection are basic diet ingredients, such as fruit, vegetables, and seafood.^4–9^ Considering that it spreads easily and commonly contaminates food samples, a biosensor which could sensitively and selectively detect NoV is desirable.

Electrochemical immunosensors demonstrate unparalleled advantages over traditional techniques, prominently characterized by their superior properties. First and foremost, these innovative sensors allow for efficient miniaturization, enhancing portability, a crucial aspect that facilitates their broader applicability.^10^ Their inherent cost-effectiveness, when compared to conventional counterparts, positions electrochemical biosensors as a practical and economically viable solution for diagnostic applications. Additionally, the heightened sensitivity of electrochemical immunosensors deserves explicit attention. This distinctive feature allows for the accurate detection of target analytes even at low concentrations, a critical capability, especially in scenarios like norovirus infections where the viral load is relatively minimal. Furthermore, the compatibility of electrochemical immunosensors with various matrices and their versatility in accommodating diverse biomolecules contribute to their significance. These sensors can be tailored to specific applications, offering a flexible platform for the detection of a wide array of analytes.^11,12^

The future of medical diagnostics, particularly in the detection of viral diseases, may relate to innovative biosensors. One of the promising elements used in the bioreceptor layer is antibodies, due to their high specificity towards the analyte to be detected.^13^ The basic factor in the fabrication of an electrochemical immunosensor is a proper preparation of a chemically modified electrode by the immobilization of a biologically active compound, an antibody, on its surface. One of the commonly used strategies is the covalent binding based on strong, covalent interactions between the surface of the modified electrode and the functional groups of the antibody, as it creates more stable and better oriented immunosensing platforms.^14–16^ Since bare electrodes do not possess proper sites for covalent bonding, it is crucial to successfully modify their surface to prepare it for protein anchoring. For this purpose, some reagents can be applied, such as glutaraldehyde, carbodiimide succinimide ester, or *N*-hydroxysuccinimide.^17^ Another often used strategy is the application of the self-assembled monolayer (SAM) method to obtain functionalized thin film rich in groups desired for the antibody immobilization.^18,19^ The result is the formation of highly organized SAMs due to semi-covalent binding between a sulfide group (*e.g.* thiols) and the noble metal surfaces (*e.g.* Au). Thiol functionalized antibodies possess high affinity to Au surfaces, and therefore they can be easily immobilized.^20^

In the advancement of electrochemical immunosensing strategies, enhancing signal amplification, sensitivity, and stability emerges as pivotal for optimizing immunosensor performance. Signal amplification within electrochemical immunosensors can be effectively realized through the incorporation of *e.g.* nanomaterials.^21^

To additionally enhance important working parameters of an electrochemical immunosensor, semi-conductive properties need to be improved. Commonly, it is achieved by the application of semi-conductive materials to the electrode surface. Nowadays, considerable focus has been devoted to nanomaterials, which could be applied in biosensors due to their exquisite sensitivity in chemical and biological sensing.^22^ A wide range of nanomaterials could be applied in the biosensor systems, however gold nanoparticles (AuNPs) have received great interest due to their many beneficial characteristics, such as high surface-to-volume ratio or high surface energy, which allow for a stable immobilization of biomolecules with simultaneous retention of their bioactivity.^23–25^ Additionally, they permit fast and direct electron transfer between a broad range of electrochemically detected analytes and electrode surface.^25^

In this study, we present an electrochemical biosensor consisting of a self-assembled monolayer on a gold electrode, modified with gold nanoparticles, a linker, and an antibody specific for the VP1 protein of norovirus based on a murine model. Scanning electron microscopy (SEM), electrochemical impedance spectroscopy (EIS), as well as cyclic voltammetry (CV) were applied for the visualization of each step of electrode preparation. The novelty of presented research lies in the preparation of a new, very stable matrix for the NoV detection, which could be used as a biorecognition part in a biosensorics device. Differential pulse voltammetry was used for the NoV detection and characterization of the immunosensor working parameters, such as linearity, sensitivity, selectivity, and accuracy. Described immunosensor is distinguished by good stability obtained by properly performed modification of the electrode surface, which gave very promising results in possible application as a future point-of-care device.

## 2. Materials and methods

### 2.1. Chemicals

l-Cysteine (Cys), gold nanoparticles (diameter 20 nm, stabilized suspension in citrate buffer), protein crosslinker 1-ethyl-3-(3-dimethylaminopropyl)carbodiimide hydrochloride (EDC), amine-reactive crosslinker *N*-hydroxysuccinimide ester (NHS), anti-norovirus (MNV-1) mouse monoclonal antibody, clone 5C4.10, Cat. No. MABF2097, 0.10 mg mL^−1^ (Ab), bovine serum albumin (BSA), norovirus, ferrocyanide, and glutaraldehyde (GA) were purchased from Sigma-Aldrich Co (Merck company). Sulfuric acid (H_2_SO_4_), citric acid (CA), NaOH, NaH_2_PO_4_, Na_2_HPO_4,_ KH_2_PO_4_, HCl, and CH_3_COOH were purchased from Chempur. NaCl, KCl, and CH_3_COONa were purchased from POCH (Part of Avantor, Performance Materials, Poland). Tris was purchased from Roche. All chemicals were of analytical grade and were used without any further purification. All buffers were prepared according to generally known, obligatory standards. Fruits, vegetables, and oysters for analysis in the food samples were obtained from the Selgros store. Bacteria: *E. coli*, *B. subtilis*, *Salmonella* spp, *E. faecalis*, were obtained courtesy of Prof. Jaroslaw Król from the Department of Pathology, Wrocław University of Environmental and Life Sciences, Poland.

### 2.2. Preparation of virus samples

#### 2.2.1 Cell culture

*In vitro* experiments were carried out using macrophage cells RAW (TIB-7, ATCC) (American Type Culture Collection, Manassas, VA, USA). Cells were grown in Dulbecco's Modified Eagle's Medium (DMEM) with non-essential amino acids with addition of 10% of fetal bovine serum (Biological Industries, Kibbutz Beit-Haemek, Israel), cultures were maintained in Starsted culture flasks (25 mL) at 37 °C in an incubator supplied with 5% CO_2_ and 95% air humidity. When the cells reached confluence of 90%, they were split using 0.25% trypsin–EDTA and distributed evenly into new flasks.

#### 2.2.2 Virus propagation

The virus used in the study was Murine Norovirus (VR-1973, ATCC) replicated in RAW cells. When the culture reached the confluence of 75–80%, the medium was removed, and the cells were washed with PBS solution with calcium and magnesium ions. After that, 3 mL of virus suspension was added to the flask. Cells with virus were incubated for 3 h at 37 °C in an incubator with 5% CO_2_. After incubation, virus suspension was removed, cell culture was washed with PBS and DMEM was added. Cells were incubated for 4 to 6 days and observed daily for the development of cythopatic effect (CPE) using inverted microscope (Olympus Corp., Hamburg, Germany; Axio Observer, Carl Zeiss MicroImaging GmbH).

### 2.3. Preparation of samples for accuracy studies

The white food matrices were selected based on specific criteria, taking into consideration factors such as potential susceptibility to norovirus contamination. The selection process aimed to encompass a variety of food items commonly associated with norovirus outbreaks. The chosen matrices included: Bilberry (*Vaccinium* L.), Lettuce (*Lactuca sativa* L.), Raspberry (*Rubus idaeus* L.), and Edible Oyster (*Ostrea edulis*) fished in France and the Netherlands. This materials were taken (5 mm × 5 mm), finely minced with a scalpel, and suspended in a measuring buffer, to which 100 μL of norovirus (1 × 10^−8^ TCID_50_) was added. A sample of each pathogen causing symptoms similar to norovirus infection (*Enterococcus faecalis*, *Salmonella* spp., *Escherichia coli*, *Bacillus subtilis*) at a concentration of 1.5 × 10–8 CFU, was mixed 1 : 1 with the virus.

### 2.4. Apparatus

Electrochemical measurements – cyclic voltammetry (CV) and differential pulse voltammetry (DPV) were performed using a potentiostat/galvanostat Autolab PGSTAT 128N with NOVA software at room temperature. Electrochemical measurements were carried out using a three-electrode system consisting of the modified and unmodified gold electrode (AuE, diameter 1.6 mm, produced by BASi, MF-2014 model) as the working electrode, an Ag|AgCl (sat. KCl) as a reference electrode, and a platinum wire as a counter electrode. Impedance spectra were recorded in a separate experiment using Gamry Interface 1010E potentiostat. A 50 mL volume electrochemical cell (BE H-Cell from redox.me) was used, containing: working electrode (AuE, diameter 1.6 mm, produced by BASi, MF-2014 model, 0.018 cm^2^ geometric area exposed to the solution), the Ag|AgCl (3 M KCl) reference electrode and platinum coil as a counter electrode. Equivalent circuit modeling was performed in ZView (SAI) software. Visualization and characterization of each step of the AuE modification was carried out with FEI Quanta 250 SEM microscope equipped with SDD Octane Elect Plus EDS detector. SEM imaging was performed in SE (secondary electron) mode with accelerating voltage of 5 kV under 10^−4^ Pa pressure. Sample tilt of *ca.* 70° and dynamic focus were used to reflect better surface morphology and topography. Real time reverse transcription polymerase chain reaction (RT-PCR) was applied for the isolation of the genetic material. The commercial Total RNA Maxi kit (A&A Biotechnology, Poland) was used to isolate the genetic material from the samples used in the study. A CFX Connet Bio-Rad thermocycler was used for the quantitative polymerase chain reaction (qPCR) reaction. The commercial SensiFAST™ Probe No-ROX One-Step kit (BIO-76005) (BLIRT, Poland) was used. For the identification of the genetic material, the following primers and probe were used based on literature.^26^MNV-F CCGCAGGAACGCTCAGCAGMNV-R GGYTGAATGGACGGCCTGMNV-TPd FAM-ATGAGTGATGGCGCA

The qPCR reaction conditions were as follows: 10 min at 45 °C and 2 min at 95 °C, followed by 45 cycles of 5 s at 95 °C and 20 s at 55 °C.

### 2.5. Fabrication of the immunosensor

**Fig. 1:**
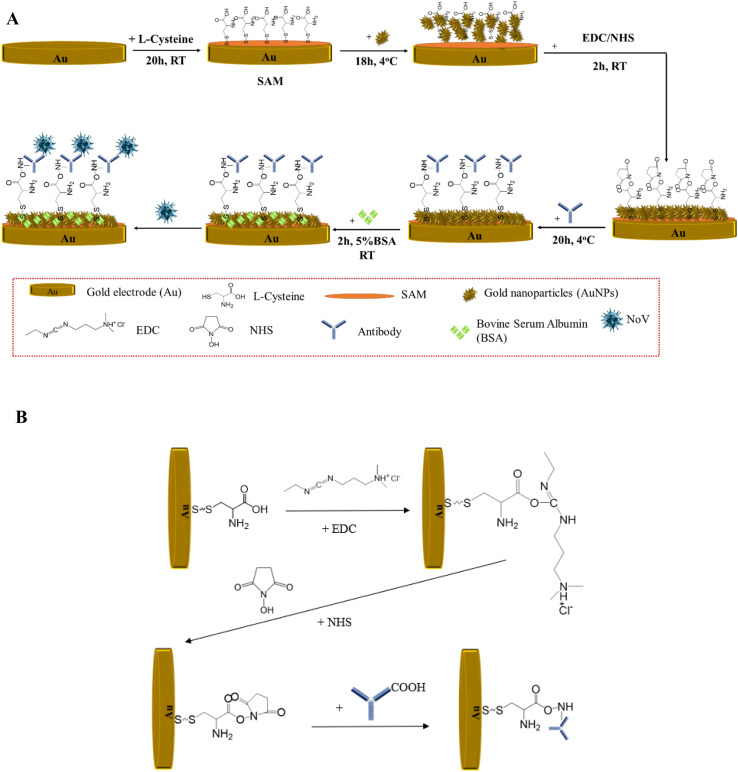
(A) Schematic preparation of the immunosensor for the NoV determination; (B) proposition of the chemical groups involved in the process of creating a stable matrix.

Preparation of the immunosensor for the detection of NoV was a multistep fabrication process and is presented in Fig. 1. Firstly, AuE was polished with diamond powder (diameter 3 μm), rinsed in ethanol and deionized water, and dried at room temperature to remove dusts and impurities from its surface. The activation of the gold electrode was performed using 0.5 M sulfuric acid for 60 cycles with a cyclic voltammetry method (potential range 0–1.4 V, until a stable voltammogram was obtained). In the first step of the proper modification, the AuE was immersed in 0.05 M l-cysteine and incubated for 20 h to spontaneously form a self-assembled monolayer, creating a film rich in thiol groups. l-Cysteine is rich in S-atom which interacts with Au-atom from electrode through Au–S binding, it has high affinity to the Au NPs as well. Next, 40 μL of gold nanoparticles were dropped onto the AuE/SAM electrode and incubated for 18 h at 4 °C. During the next step, 20 μL EDC/NHS was added onto the surface for 2 h at RT. Application of EDC/NHS results in the creation of a well-oriented monolayer and allows a better antibody/antigen interaction.^27^ The last step was the immobilization of the antibody, which was diluted 1 : 5000, according to the literature,^28^ and 20 μL was dropped onto the AuE/SAM/AuNPs/EDC/NHS electrode for 20 h at 4 °C. Since the amide bond created between the amino group of the NHS and the carboxyl group of the protein is strong, the antibody does not detach from the electrode surface during measurements. In the end, 0.5% bovine serum albumin (BSA) was incubated on the electrode surface area for 2 h to block inactive groups and non-specific binding sites. The detection was based on the specificity of the virus surface protein VP1 to the antibody.

The electrode was rinsed with 0.1 M PBS (pH 7.0) and 10 mM pH 8.0 Tris–HCl buffers for 10 minutes each and with deionized water after each step of the modification process to wash unbound molecules from the electrode surface and remove impurities or any cross-linker byproducts.

Each step of the modification was checked using CV and EIS analysis. SEM was adopted for the visualization of the surface of the gold electrode after modification. Prepared electrode was stored at 4 °C when not in use.

### 2.6. Electrochemical procedure of the norovirus analysis

A typical three-electrode 10 mL cell, including the above-mentioned gold working electrode, platinum counter electrode, and Ag|AgCl reference electrode, was used for the CV and DPV measurements. The CV method was applied for the visualization of each step of the electrode modification towards 5 mM ferro/ferricyanide redox couple in 0.1 M KNO_3_ (as the supporting analyte). The CV scans were recorded in a potential range from −0.4 V to 0.6 V *vs.* Ag|AgCl electrode for 3 cycles each, at a scan rate of 50 mV s^−1^. In addition, potentiostatic EIS was performed at open circuit potential (*E*_OC_) in 0.1 M KNO_3_ (supporting electrolyte) solution containing 5 mM [Fe(CN)_6_]^3−/4−^ (1 : 1) with a resolution of 10 pts per dec in a frequency range from 100 kHz to 0.01 Hz and ac signal of 10 mV (rms).

For the characterization of the immunosensor's working parameters, the differential pulse voltammetry technique was used. DPV for a range of dilutions (10^−1^–10^−10^) of the NoV (1 × 10^−8^ TCID_50_ – tissue culture infective dose) was performed in the potential range of 0–0.6 V *vs.* Ag|AgCl and with the amplitude of 50 mV. To test the immunosensor's ability to work in open-air conditions and at room temperature, all electrochemical measurements were performed under such conditions. Samples of the NoV in the concentration range of 1 × 10^−9^ to 1 × 10^−18^ TCID_50_ were prepared by serially diluting norovirus samples in 0.1 M PBS buffer at pH 7.0 containing 0.1 M KCl (supporting electrolyte).

### 2.7. Accuracy and stability tests

Common food samples coming from lettuce, bilberry, raspberry, and oysters (from France and Holland), and samples from widespread bacterial pathogens (*E. coli*, *B. subtilis*, *Salmonella* spp., *E. faecalis*), prepared as described in Section 2.3, were infected with the NoV and studied for the accuracy of described immunosensor. Mentioned species were mixed each time with the NoV solutions in a volume ratio of 1 : 1. DPV analysis was then applied in the potential range of 0–0.6 V *vs.* Ag|AgCl and signals were compared to the one obtained from the NoV sample.

The stability test of the immunosensor was conducted 2 weeks after the electrode preparation using the CV analysis with AuE/SAM/AuNPs/EDC/NHS/Ab/BSA in the presence of 5 mM ferro/ferricyanide redox couple in 0.1 M KNO_3_ for 60 cycles in a potential range of −0.4–0.6 V *vs.* Ag|AgCl reference electrode with the scan rate of 50 mV s^−1^.

### 2.8. Ethical statement

This research did not involve human or animal samples.

## 3. Results and discussion

### 3.1. Characterization of the electrochemical and microscopic changes during the construction of the immunosensor

The multi-step process of the electrode modification was characterized by SEM, EIS, and CV. The immobilization of the agents on the electrode surface was confirmed after each step.

**Fig. 2:**
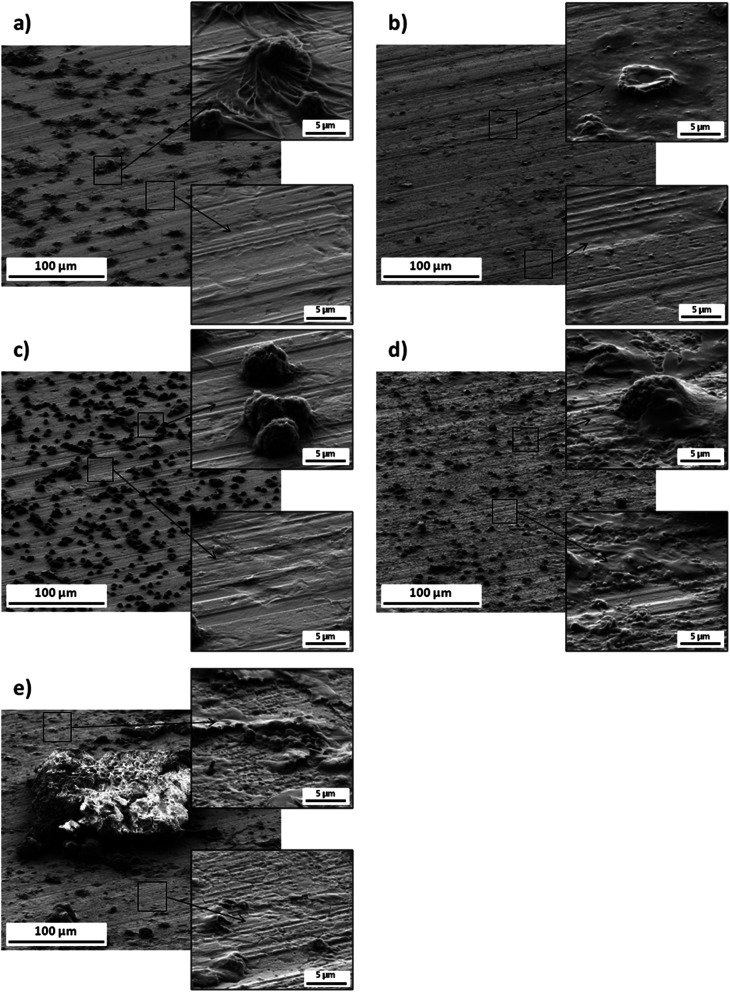
SEM images of surface morphology of the electrode modified with: SAM (a), AuNPs (b), SAM/AuNPs (c), SAM/AuNPs/EDC/NHS (d), SAM/AuNPs/EDC/NHS/antibody (e).

SEM was used to monitor the changes in surface morphology after each modification step. The first step led to the formation of SAMs with a fairly diverse morphology, *i.e.* the surface was dotted with spheroidal and randomly distributed clusters (few micrometers in size) embedded in the l-cysteine layer (Fig. 2a). For comparison, the AuNPs deposited on the raw gold substrate formed a generally uniform layer with scattered very fine agglomerates and relatively large and flat clusters with a diameter of several micrometers (Fig. 2b). Combining these two steps together resulted in a denser, than in Fig. 2a, surface coverage with several micrometer nodules with a flocculent and (probably) porous structure (Fig. 2c). Addition of EDC/NHS layer caused that the surface after SAM/AuNPs modification was covered with a quite differentiated layer (Fig. 2d). Namely, the nodules were covered (the smaller ones) or partially embedded (the larger ones) in a fairly smooth and continuous EDC/NHS layer. Outside the EDC/NHS clusters, the layer was discontinuous, of varying thickness, and granular in structure (Fig. 2d). Immobilization of the antibody (Fig. 2e) resulted in fine granularity in places where the EDC/NHS layer was the thinnest. In addition, few, flat clusters with sizes exceeding 100 micrometers were visible – probably formed after dropping the antibody onto the surface.

**Fig. 3:**
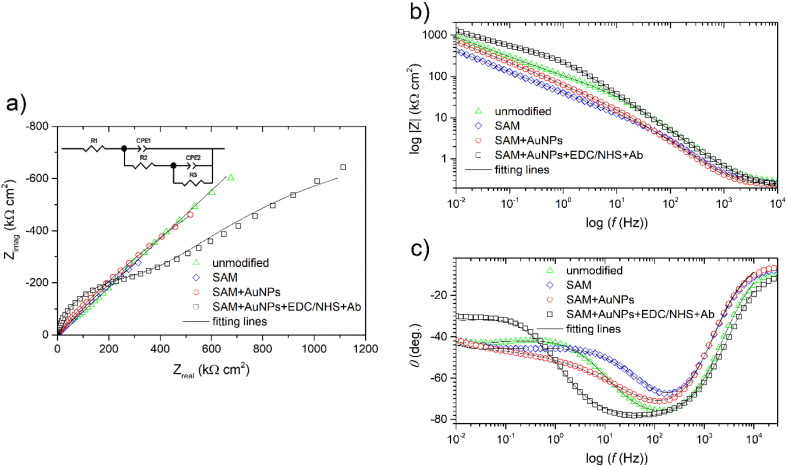
Nyquist (a) and Bode (b and c) representations of the impedance spectra recorded for unmodified and modified gold electrodes at *E*_OC_ after 30 min of exposure in a solution of 0.1 M KNO_3_ with 5 mM [Fe(CN)_6_]^3−/4−^. Solid fitting lines refer to Model 2 from Fig. 4b.

Impedance spectra recorded for all samples with a modified surface are characterized by a similar shape that slightly differs from that of unmodified Au electrode (Fig. 3a–c). In the Nyquist plot (Fig. 3a), one time constant can be distinguished in the medium frequency range. A further increase in impedance, starting from a few Hz towards mHz, can be interpreted in two ways. On the one hand, it may be a fragment of the second semicircle. On the other hand, this fragment is almost perfectly inclined (for the first three samples only) at an angle of 45° and may indicate the existence of diffusion constraints. Regardless of the interpretation, the impedance characteristics are similar (in shape) to those in the literature.^29–31^ While the presence of a second time constant is hard to see on the Nyquist plot (Fig. 3a), the second maximum of the phase angle could be found on the Bode plot at about 0.1 Hz (Fig. 3c). It is especially visible for the electrodes after modification. Apart from that, all samples reach rather high impedances (Fig. 3b).

**Fig. 4:**
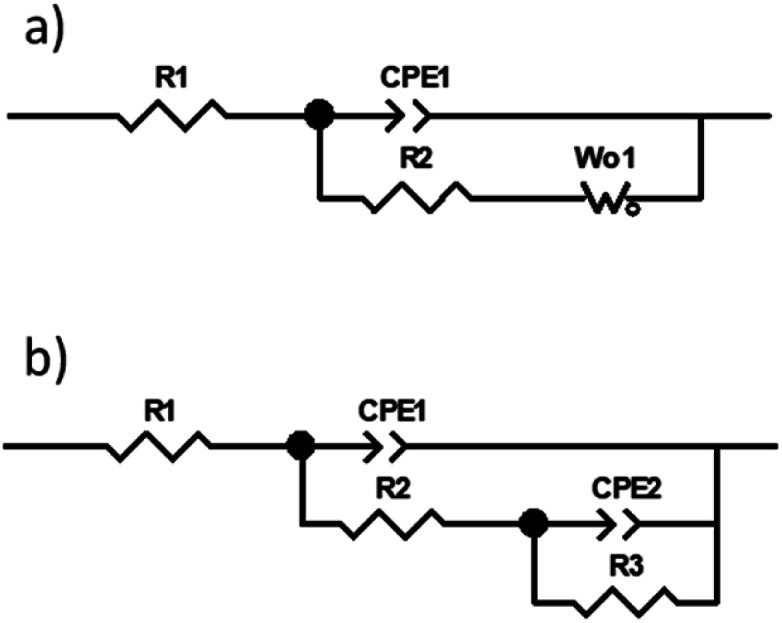
Electric equivalent circuits used for fitting the experimental impedance spectra recorded for both modified (a) and unmodified (b) gold electrodes.

The use of the simplest model with one time constant containing an open Warburg element connected in series after the resistor was initially considered (Fig. 4a). In this circuit the physical sense of the elements could be as follows: *R*_1_ – electrolytic solution resistance, CPE_1_ and *R*_2_ – the capacitance and resistance of the charge transfer reaction, *W*_o_ – Warburg diffusion element, where: *R* reflects diffusion impedance, *T* reflects time of diffusion of the particle through layer thickness and *P* is the *W*_o_ exponent.

Quite good fit (chi square *ca.* 4.3 × 10^−5^–2.5 × 10^−4^) and low residual errors were obtained. Fitting results, shown only for exploratory and illustrative purposes, are summarized in Table 1, without delving into the physical meaning of the elements.

**Table 1:** Fitting results for impedance spectra recorded on modified and unmodified Au electrodes in 0.1 M KNO_3_ with 5 mM [Fe(CN)_6_]^3−/4−^ solution obtained for a model from Fig. 4a^a^

Sample	*R*_1_ (Ω cm^2^)	CPE_1_–*T* (Ω^−1^ cm^−2^ s^P^)	CPE_1_–*P*	*R*_2_ (kΩ cm^2^)	*W*_o_–*R*	*W*_o_–*T*	*W*_o_–*P*
AuE	269 (0.7)	4.90 × 10^−7^ (0.8)	0.93 (0.1)	44.77 (1.5)	2.79 × 10^6^ (4.5)	164 (9.7)	0.49 (0.3)
AuE + SAM	254 (0.5)	7.49 × 10^−7^ (3)	0.94 (0.4)	7.44 (4)	1.16 × 10^6^ (4.5)	118 (9.6)	0.51 (0.3)
AuE + SAM + AuNPs	238 (0.5)	7.89 × 10^−7^ (3.8)	0.94 (0.5)	8.01 (10)	1.95 × 10^6^ (4.3)	124 (9.5)	0.49 (0.3)
AuE + SAM + AuNPs + Ab	226 (0.7)	6.10 × 10^−7^ (1)	0.90 (0.2)	304.9 (3.6)	3.42 × 10^6^ (32)	268 (81)	0.49 (2.3)

a Values in brackets are the % error.

Fitting was repeated, but the circuit was modified to the one consisting of two time constants connected in parallel (Fig. 4b). Using this physical model, the best fit (chi square *ca.* 3–9 × 10^−5^) and low residual errors were obtained in this work. Fitting results are summarized in Table 2, and the fitting lines visible on Nyquist and Bode plots in Fig. 3a–c are related directly to these results. In this circuit, the physical sense of the elements was adopted, according to ^29–31^, as follows: *R*_1_ – electrolytic solution resistance, CPE_1_ and *R*_2_ – the frequency response of the charge transfer process, while CPE_2_ and *R*_3_ – the frequency response of the diffusion process. Normally, if *P* = 0.8–0.9, then CPE_2_ would describe some real capacitance. However, here *P* ∼ 0.5 so this constant phase element behaves rather like a Warburg element. This assumption is supported by the previous fitting using the circuit from Fig. 4a.

**Table 2:** Fitting results for impedance spectra recorded on modified and unmodified Au electrodes in 0.1 M KNO_3_ with 5 mM [Fe(CN)_6_]^3−/4−^ solution obtained for a model from Fig. 4b^a^

Sample	*R*_1_ (Ω cm^2^)	CPE_1_–*T* (Ω^−1^ cm^−2^ s^P^)	CPE_1_–*P*	*R*_2_ (kΩ cm^2^)	CPE_2_–*T* (Ω^−1^ cm^−2^ s^P^)	CPE_2_–*P*	*R*_3_ (MΩ cm^2^)
AuE	269 (0.3)	4.94 × 10^−7^ (0.9)	0.93 (0.1)	45.95 (1.9)	4.35 × 10^−6^ (0.3)	0.50 (0.6)	27.5 (22)
AuE + SAM	252 (0.8)	8.14 × 10^−7^ (1.8)	0.93 (0.2)	8.98 (2.3)	9.49 × 10^−6^ (0.7)	0.53 (0.4)	2.91 (6)
AuE + SAM + AuNPs	237 (0.2)	8.46 × 10^−7^ (1.5)	0.94 (0.2)	11.24 (3.7)	5.32 × 10^−6^ (0.2)	0.51 (0.3)	5.04 (4.6)
AuE + SAM + AuNPs + Ab	224 (0.2)	6.29 × 10^−7^ (0.5)	0.90 (0.1)	377.2 (1.5)	3.89 × 10^−6^ (1.9)	0.60 (1.9)	2.49 (5.5)

a Values in brackets are the % error.

Table 2 shows that the deposition of SAM layer reduced the resistance of *R*_2_ five times, below 10 kΩ cm^2^, but more importantly, reduced the resistance *R*_3_ by almost one order of magnitude. If one associates *R*_2_ with electron transfer and *R*_3_ with diffusion constraints, then their large decrease would confirm that one has achieved the goal of this sub-step of modification. The next step, the deposition of AuNPs on the surface, caused only a slight increase in both resistances (Table 2). It can therefore be confirmed that surface functionalization with gold nanoparticles also facilitates electron transfer between detected analytes and Au electrode surface, in accordance with the literature.^25^ A significant change was caused by the immobilization of the antibody. Although *R*_2_ has increased more than thirty times, *R*_3_ remained at a low level, in the order of 2.5 MΩ cm^2^ (Table 2). This means that although the electron transfer resistance has increased (because the antibody inhibited electron transfer), the diffusion constraints have decreased slightly. In addition, the CPE_1_–*P* parameter slightly decreased, which would suggest greater heterogeneity (distribution of reaction rates) within the surface. All these findings would confirm that the antibodies have been successfully grafted onto the surface of the modified electrode.

**Fig. 5:**
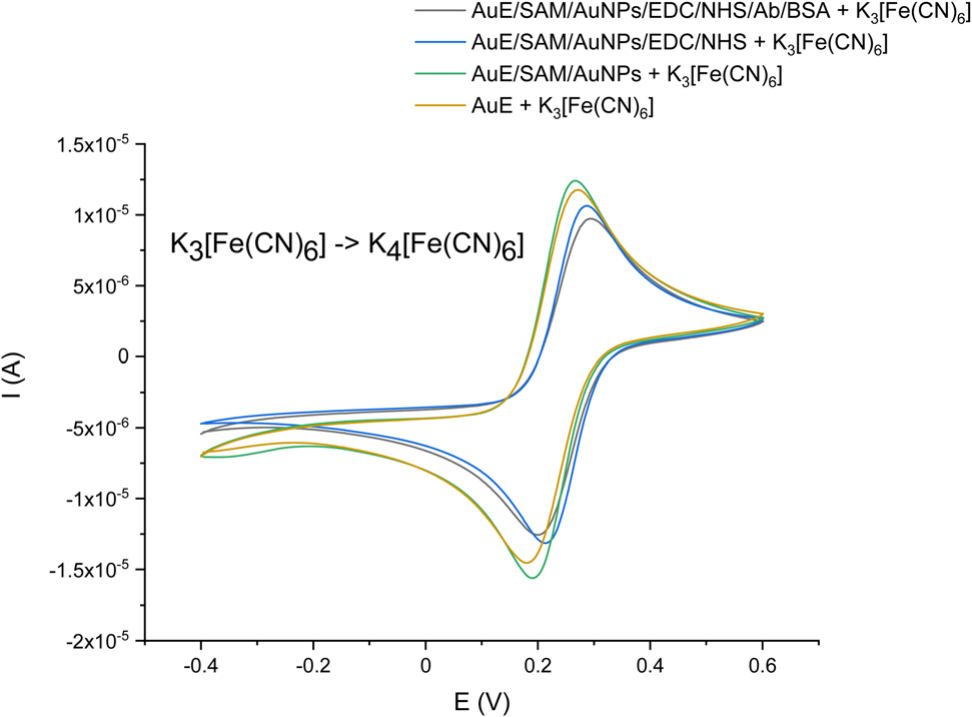
CV voltammogram presenting the preparation of the electrochemical immunosensor for the NoV detection to 5 mM ferri/ferrocyanide redox probe, KNO_3_ used as a supporting electrolyte; potential range: −0.4–0.6 V; scan rate: 50 mV s^−1^; *vs.* Ag|AgCl.

Using this physical model, a CV was also used to characterize the gold electrode after each assembly step. As can be observed in Fig. 5, the cyclic voltammogram of ferri/ferrocyanide redox couple demonstrated reversible behavior on a bare Au electrode, with a peak-to-peak separation of *c.a.* 30 mV (Fig. 5, gold curve). When AuE was modified by the immobilization of SAM and AuNPs onto its surface, the peak current increased noticeably (Fig. 5, green curve) because of an exquisite electron transfer performance of AuNPs.^32^ As expected, the immobilization of the biorecognition element for the NoV detection (the antibody) onto the electrode surface caused a current signal decrease, with peak-to-peak separation *c.a.* 60 mV (Fig. 5, black curve). The covalent assembly of the antibody onto the electrode could form a closely packed film which passivates the electrode, hindering the transfer of electrons between the redox probe and the electrode. This phenomenon was observed in numerous other studies.^30,33,34^

Results described above corroborate that all molecules were successfully used to modify the AuE surface, and the immunosensor could be applied for the VP1 determination.

**Fig. 6:**
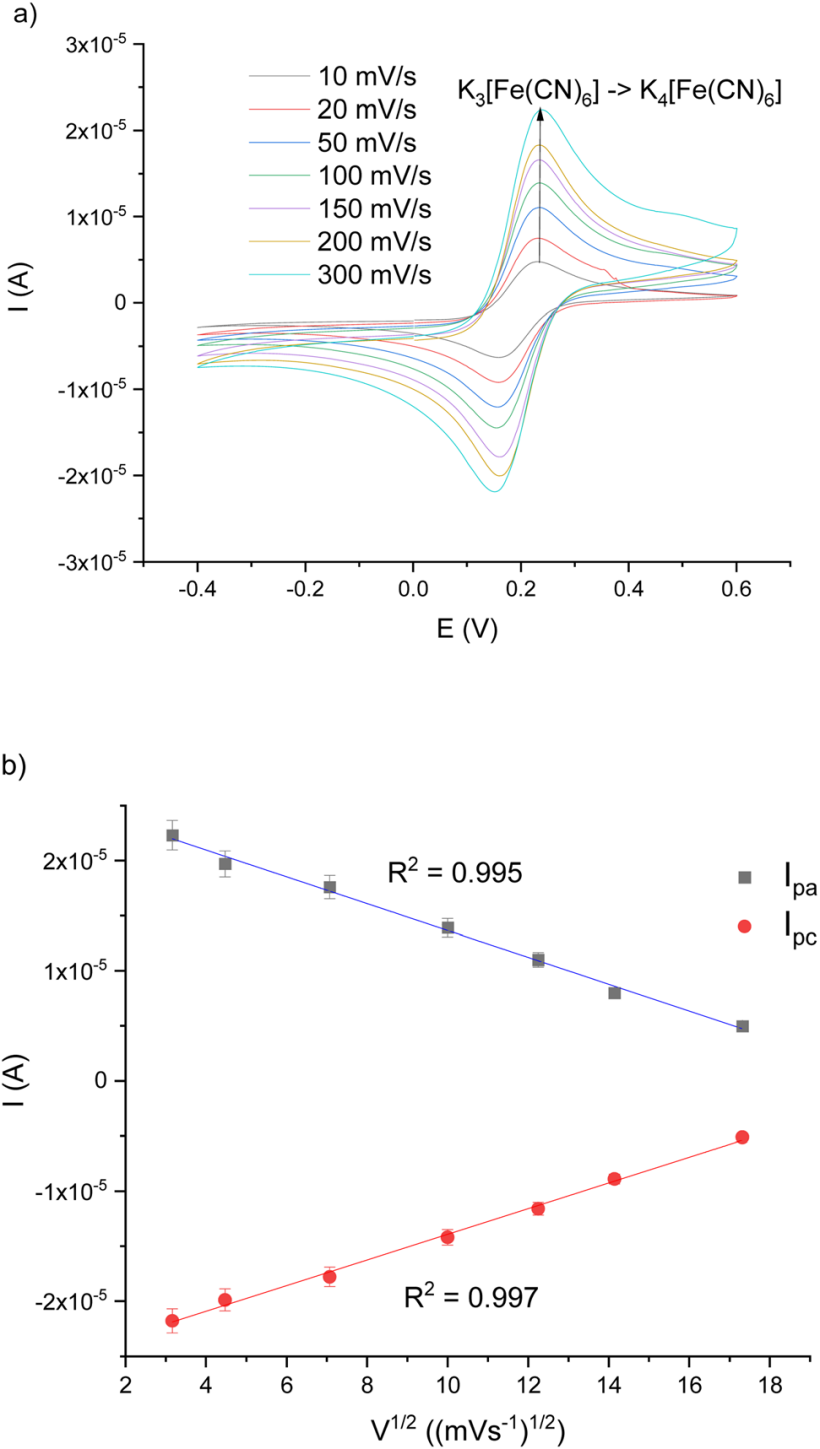
(a) CV-voltammogram showing AuE/SAM/AuNPs/EDC/NHS/Ab/BSA in 5 mM ferri/ferrocyanide redox probe and 0.1 M KNO_3_ at different scanning rates (10–300 mV s^−1^); potential range: −0.4–0.6 V; *vs.* Ag|AgCl; (b) linear relationship between scan rate and current (*I*_pc_ – red line; *I*_pa_ – blue line).

Furthermore, the scan rate and the correlation between the current of anodic/cathodic peak were also investigated at every step during the immunosensor fabrication. CV has been performed in the same potential range at different scan rates (10–300 mV s^−1^). As can be observed in Fig. 6a, current signals obtained from both cathodic and anodic peaks (*I*_pc_, *I*_pa_, respectively) increase along with the scan rates. In combination with the results obtained from the relationship of *I*_pc_ and *I*_pa_ values *vs.* square root of scan rates (*v*^1/2^), presented in Fig. 6b, which shows good linearity (*R*^2^ = 0.995 for *I*_pa_ and *R*^2^ = 0.997 for *I*_pc_), it may suggest that the electrochemical process of the interface for the immunosensor was a diffusion-controlled process.^35^ Because of that, results obtained in the next step are only from the tested VP1 protein of NoV.

### 3.2. Analytical performance of the immunosensor

For the characterization of the working parameters of the immunosensor, the DPV technique was used.

**Fig. 7:**
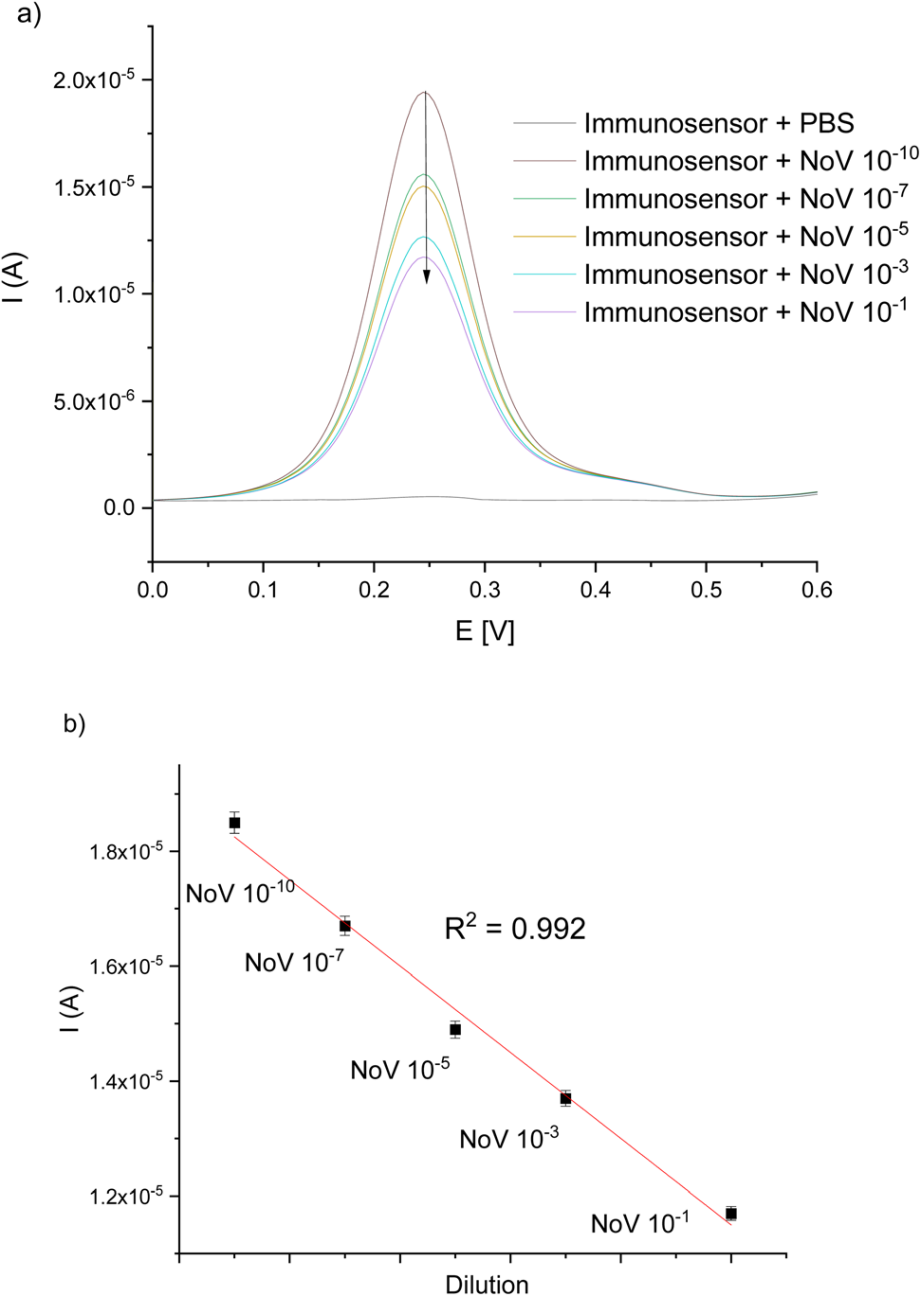
(a) Representative DPV-scans for different NoV dilutions in the potential range of 0–0.6 V *vs.* Ag/AgCl; (b) linear relationship between the current and NoV dilution.

As can be seen in Fig. 7a, obtained DPV peaks were inversely proportional to the exposed concentrations of NoV, due to the fact that binding of the VP1 protein of NoV to the antibody causes an increase in resistance and therefore a decrease in current values.^30^ The corresponding calibration curve, shown in Fig. 7b, represents good linearity in the investigated range (1 × 10^−9^–1 × 10^−18^ TCID_50_) with *R*^2^ = 0.992. The detection limit (LOD) was the lowest concentration of virus detected by presented method – 1 × 10^−18^ TCID_50_.

Regeneration of the immunosensor was done by soaking the platform in 200 mM KSCN solution^36^ for 4 min and then washing it with doubly distilled water and PBS solution for 30 seconds.

Some examples of electrochemical immunosensors and biosensors for NoV detection can be found in recent literature. Hong *et al.* reported biosensor based on concanavalin-A (Con-A) immobilized onto a gold electrode to detect human NoV, applying an electrochemical transducer.^31^ The developed biosensor gave a response of NoV detection after approx. 1 h and presented good parameters of sensitivity and selectivity. Accuracy test provided in lettuce gave a very good response with a detection limit of 60 genomic copies per mL. Another electrochemical approach presented by Wang *et al.* was based on the utilization of a modified aptamer in the biorecognition part for murine norovirus detection (as an alternative to human NoV).^37^ Gold electrode was modified by a thiolated aptamer AG3 and specific binding to murine NoV. The researchers applied the cyclic voltammetry method to characterize the aptamer binding and square wave voltammetry (SWV) was employed to check the NoV capture. Such immunosensor gave a sensitive and fast response and was simple to prepare. Chand and Neethirajan described a microfluidic chip consisting of screen-printed carbon electrodes and polydimethylsiloxane for the electrochemical detection of human NoV.^38^ Silica magnetic beads were used to fill the chip for concentrating the virus samples and also for the filtration of clinical samples. The biorecognition part was prepared as follows: carbon electrode was modified with gold nanoparticles and thiolated streptavidin, onto which the human NoV-specific aptamers labelled with ferrocene (as redox probes) and biotin were immobilized. Scientists conducted their research using differential pulse voltammetry and obtained the detection limit equal to 100 pM.

### 3.3. Tests in food and bacteria samples, reproducibility and stability studies

**Fig. 8:**
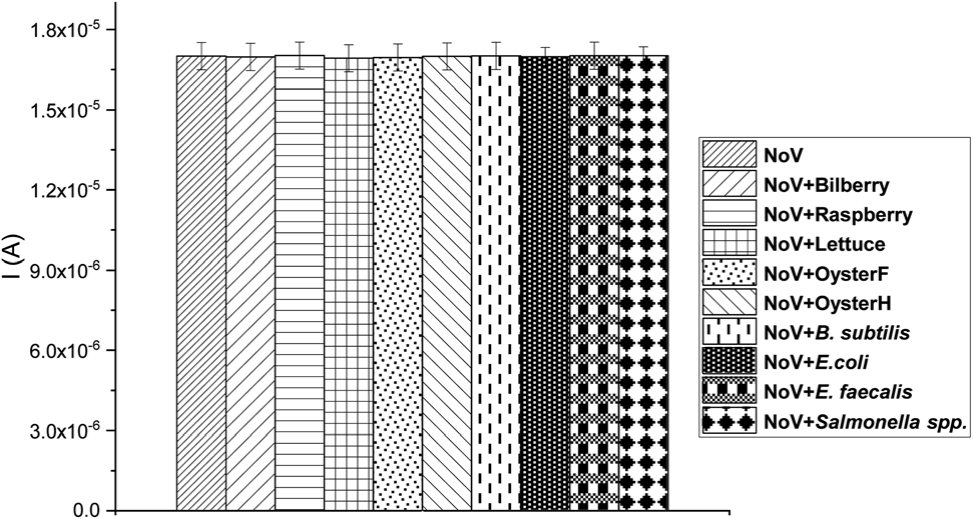
Recovery test of the immunosensor for NoV.

The selectivity, accuracy, and stability are one of the most important parameters characterizing biosensors. Described here immunosensor for the NoV detection should be suitable for medical diagnostic purposes. To test the accuracy of the constructed immunosensor, a few common food samples and bacteria species were infected with NoV an DPV was applied. Obtained current value of 1 × 10^−8^ TCID_50_ NoV was compared to the current signal obtained from the infected, investigated samples. As can be observed in Fig. 8, all analyzed species showed an excellent recovery value, only very slight (<3%) changes are visible in the current response in detected food/bacteria samples infected by NoV in comparison with NoV. Presented results clearly show that the proposed immunosensor can be successfully used for the determination of NoV in diagnostics and food quality investigations.

The reproducibility of the Au/SAM/AuNPs/EDC/NHS/Ab/BSA immunosensor for the NoV determination was tested by the DPV method by checking the current of the same concentration of NoV (1 × 10^−8^ TCID_50_) with four, modified in the same way, gold electrodes. The average current was calculated as 1554 μA. The results exhibited a 5.12% relative standard deviation (RSD), showing very good reproducibility.

**Fig. 9:**
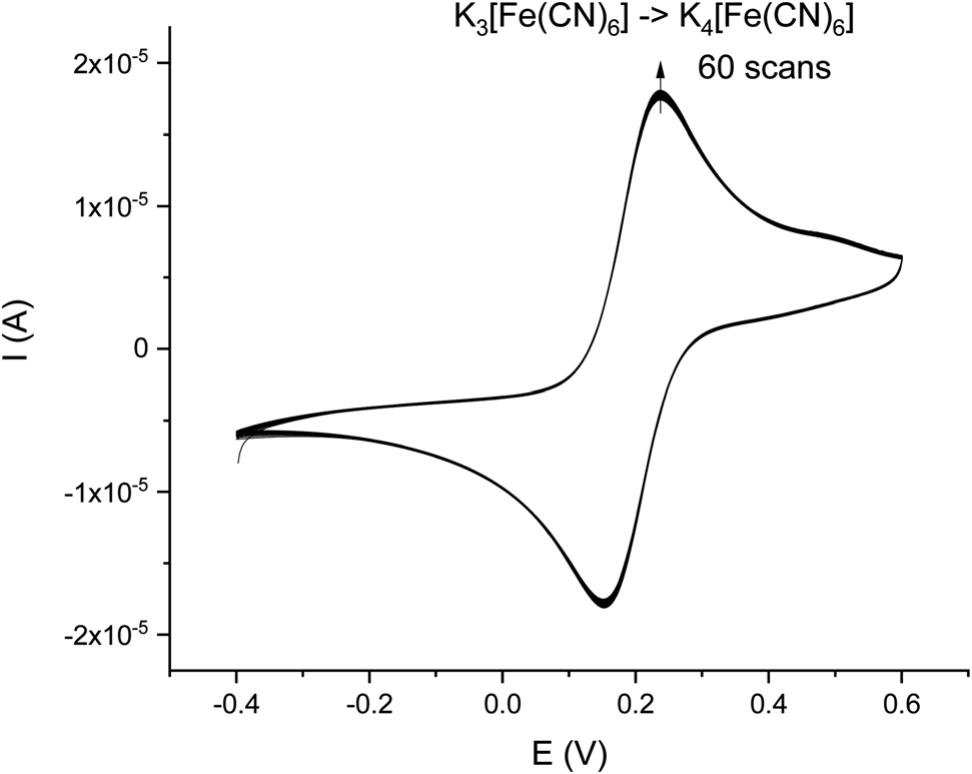
Representative CV-scans of Au/SAM/AuNPs/EDC/NHS/Ab/BSA in the presence of 5 mM Fe(CN)_6_^3−^/Fe(CN)_6_^4−^ in 0.1 M KNO_3_ after 2 weeks. Potential range: −0.4–0.6 V, scan rate 50 mV s^−1^, *vs.* Ag|AgCl electrode, 60 cycles.

Good, long-term stability is one of the issues biosensors are facing. The stability was tested after storing the immunosensor at 4 °C (humid conditions) for 2 weeks after all experiments. The stability test was carried out using the CV method (Fig. 9). The average electrical current decreased in comparison with the original signal by approx. 4%, which shows that the constructed immunosensor is characterized by very high stability, possibly resulting from a very strong and well-prepared modified electrode for the Ab immobilization.

### 3.4. RT-PCR

**Fig. 10:**
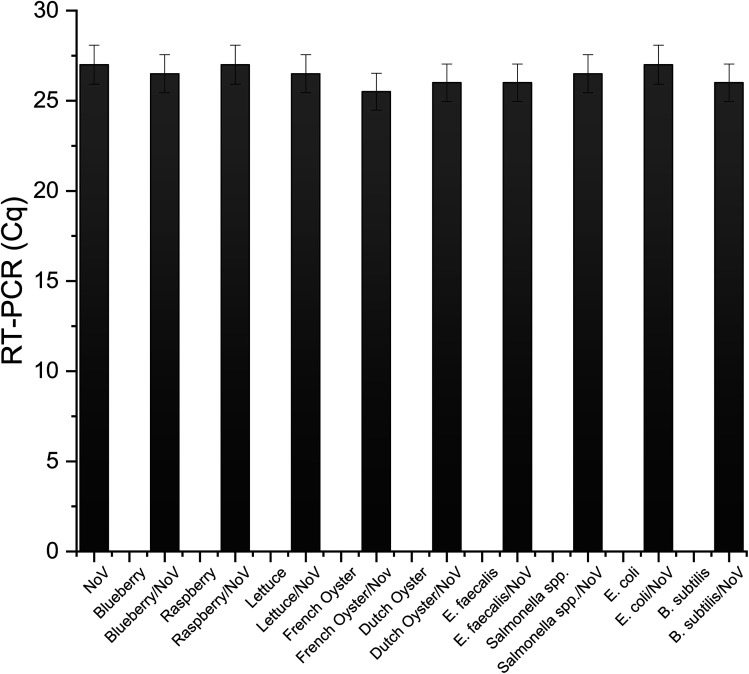
Reader tested samples submitted for RT-PCR analysis.

Laboratory mice are most commonly infected with mouse norovirus and various effects of infection have been reported.^39^ To confirm infection, tests based on RTq-PCR (real-time reverse transcription polymerase chain reaction) are often used, RT-LAMP (isothermal amplification *via* a reverse transcription loop).^40,41^ Due to the lack of a robust tissue culture system for human norovirus, mouse norovirus are often used in many studies,^42^ and still for the diagnosis of norovirus infection, the most common method is RT-qPCR, however, it is very time-consuming and requires specialized personnel and equipment, which is why other, simpler methods are researched.^30^ In this study, RT-qPCR has been used as a reference method, as an additional confirmation for the electrochemical measurements of biosensor's accuracy.^43^ As can be observed in the Fig. 10, obtained results from RT-qPCR are almost identical with selectivity tests obtained from described here electrochemical biosensor.

Western blot is used in research to separate and identify proteins. In scientific studies of human as well as mouse norovirus, the western blot technique is frequently used.

**Fig. 11:**
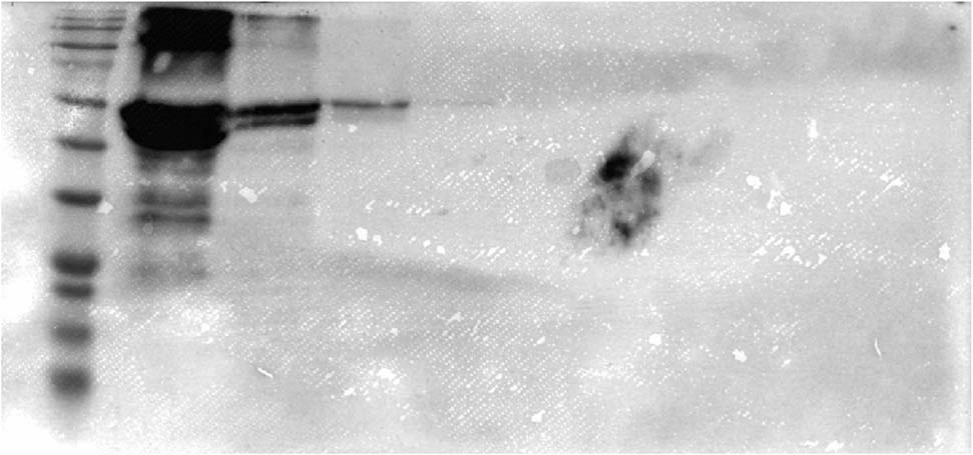
Dilution series of norovirus detected by western blot technique (1st well contained molecular ladd 3-colour prestained protein marker (10–245 kDa) blirt, the rest of the wells contained mouse norovirus at a range of dilutions from 10^−1^ to 10^−8^).

The created sensor detected the VP1 protein of mouse norovirus, and to confirm the presence of this protein in the sample, a number of dilutions were tested by western blot technique. In the experiment, 3 well visible bands and one faintly visible band were observed (Fig. 11). All striations are located at 70 kDa, which corresponds to the size of the VP1 protein.

## 4. Conclusions

In this paper, the electrochemical immunosensor using gold nanoparticles for NoV detection was constructed. AuNPs were utilized for the enhancement of obtained electrical signal and EDC/NHS allowed the creation of a well-oriented monolayer, which results in better antibody/antigen interaction. An antibody specific to the surface protein of the VP1 virus was the base of the measurement system. Binding of the VP1 protein to the antibody caused a decrease in the current, which was proportional to the concentration of NoV sample. The detection limit was 1 × 10^−18^ TCID_50_. Designed immunosensor exhibited good performance, selectivity, and sensitivity. To our knowledge, this study is the first report with application of SAM/EDC-NHS, and an antibody as a platform on a gold electrode surface for NoV detection. What is more, the described system showed excellent recovery values, good linearity, and long-term stability, which is essential for the construction of bio-devices for point-of-care testing or fast analytical devices. Future research should focus on a thorough understanding of the VP1 protein capture mechanism, which may result in the construction of a real chip for rapid diagnosis. It is also important to thoroughly optimize the system in the context of real biological samples (like feces).

## Data availability

There is an availability of sharing data and materials, after request.

## Conflicts of interest

The authors declare no conflicts of interest regarding the content presented in this article.
